# A Comparative Study on the Diagnostic Value of CTA and MRA in Anterior Dislocation of Shoulder

**DOI:** 10.1155/2022/9461236

**Published:** 2022-04-11

**Authors:** Junhua Wu, Tao Zhang, Xuxue Li, Qian Dan, Dezhou Zhang

**Affiliations:** Department of Radiology, Sichuan Provincial Orthopedic Hospital, Chengdu, Sichuan 610041, China

## Abstract

**Objective:**

To investigate the diagnostic value of CTA and MRA in anterior dislocation of shoulder.

**Methods:**

The detection of inferior glenohumeral ligament injuries, anterior inferior labrum injuries, and bone and cartilage injuries by the two examination procedures was observed and compared with the results of arthroscopy or surgery on patients referred to our hospital owing to anterior dislocation of shoulder*. Results.* A total of 36 patients with shoulder injuries were gathered for this study. 32 cases with anterior inferior labrum tearings (27 cases detected by CTA and 30 cases by MRA), 24 cases with inferior glenohumeral ligament tearings (14 cases detected by CTA and 22 cases by MRA), 24 cases with inferior glenohumeral ligament tearings (14 cases detected by CTA and 22 cases by MRA), and 24 cases with inferior glenohumeral ligament tearings (14 cases detected by CTA and 22 cases by MRA) were detected. There were 30 bone and cartilage injuries, including 18 fractures (CTA identified 18), 10 bone contusions (CTA detected 0), and 5 cartilage damage (CTA detected 0) (CTA detected 0, MRA detected 5).

**Conclusion:**

The detection rate of MRA is better than that of CTA for inferior glenohumeral ligament injuries. For anterior inferior labrum injury, the detection rate of CTA and MRA was similar. CTA is more conducive to the detection of fracture blocks, while MRA is more advantageous for the observation of bone contusion and cartilage damage.

## 1. Introduction

The most prevalent type of shoulder damage (approximately 95 percent) is anterior dislocation [1]. The importance of accurate preoperative imaging assessment in the selection of surgical procedures and prognosis cannot be overstated. Due to its benefits in fracture display, MRI has been widely employed in clinic due to its high soft tissue resolution, and CT examination plays an important role in preoperative evaluation of shoulder joint damage. By injecting contrast agent into the articular cavity to open the capsule, direct arthrography of the shoulder can better show joint capsule and brachial ligament injury, labrum injury, bone, and cartilage damage. Currently, direct arthrography of the shoulder joint mainly includes CT arthrography and MR arthrography. In this study, CTA and MRA were performed on 36 patients admitted to our hospital for anterior dislocation of the shoulder, so as to explore the best preoperative imaging methods for patients with anterior dislocation of the shoulder.

## 2. Materials and Methods

### 2.1. The General Information

A total of 36 patients with anterior shoulder dislocation who visited our hospital from January 2018 to June 2020 were collected. The patients were informed of the significance and risks of the relevant examination and signed the informed consent. This study was reviewed and approved by the hospital ethics Committee. Among them, there were 27 males and 9 females, aged from 16 to 45 years old, with an average age of (24 ± 6) years, and a course of 1 to 32 months, with an average course of (5 ± 2) months. Among them, 21 cases were chronic repeated dislocation with no obvious pain or swelling of the shoulder joint and limited movement to varying degrees. The fear test of anterior instability of the shoulder joint was all positive. 15 cases were acute single dislocation with the main clinical symptoms of shoulder joint pain, swelling, and limited movement. All the 36 patients underwent CT arthrography and MR arthrography, 28 underwent shoulder arthroscopy exploration and repair, and 8 underwent shoulder joint open surgery, with the findings of arthroscopy and open surgery as the gold standard.

### 2.2. Procedure

A 20-ml empty needle was used to prepare the contrast agent: 0.25 ml gadolinitic dextrine, 5 ml iodixanol, 5 ml lidocaine, and 5 ml 0.9% normal saline.

For CT arthrography, Siemens SOMATOM Definition AS 64 row multislice spiral CT from Germany was chosen. The patient was supine and had a forward-facing head. To ensure that the contrast agent injection into the articular cavity by fast spiral scanning, scanning parameters were as follows: voltage 120 kv, current 210-240 ma, collimating width 1.5 mm, and 0.75 1.0 mm raw data, which obtained thin-layer transverse images and spread to Siemens image postprocessing workstation for 3 d reconstruction.

For MR arthrography, after CT arthrography, the patient was rotated and abducted the shoulder joint three times, and then, MR arthrography was performed. MR angiography was performed using GE 1.5 T Signa explorer magnetic resonance scanner. Patients were randomly assigned to supine position and shoulder coil and underwent proton density-weighted fat suppressor image (PDWR-FS,TR/TE = 2517/58 ms), and T1-weighted image (T1WI,TR/TE = 450/13 ms), and T1-weighted fat suppressor image (T1wi-FS,TR/TE = 440/11 ms); sagittal T1WI-FS, horizontal T1WI-FS, and PDWR-FS were scanned; and the layers were 4 mm thick and 1 mm spaced.

### 2.3. Image Analysis and Statistics

All the image data were reviewed by 2 experienced MRI diagnostic physicians, and the osseous structure and soft tissue changes of the shoulder joint were carefully observed and analyzed, and the inconsistency of diagnosis was solved by negotiation. The main contents of observation include: (1) inferior glenohumeral ligament; (2) anterior lower lip; and (3) bone and cartilage. Observe the display of the above structures in each sequence and each azimuth, and record the imaging abnormalities.

SPSS17.0 software package was applied, and *X*^2^ test was used for statistical analysis. *P* < 0.05 was considered statistically significant.

## 3. Results

In this group, 36 patients had 36 affected shoulders. With the exception of bone contusion (MRI is the gold standard for the diagnosis of bone contusion), the results of shoulder arthroscopy were the gold standard for all the other lesions (Table 1).

### 3.1. Inferior Glenohumeral Ligament Injury

**Figure 1:**
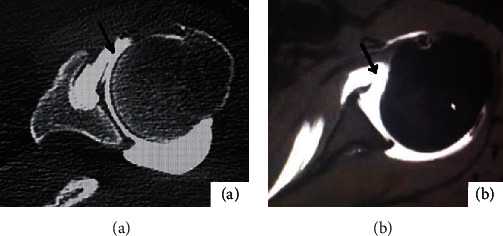
Female, 24 years old, anterior dislocation of left shoulder for 5 months. (a) shows the bone window image on the horizontal axis of CTA of the left shoulder; as shown by the black arrow, anterior inferior glenohumeral ligament was torn, and part of contrast medium was leaked. (b) shows the T1WI-FS image in the transverse axis of the MRA of the left shoulder. The black arrow shows that the anterior ligament of the inferior glenohumeral ligament is torn, and the broken end is retracted.

**Figure 2:**
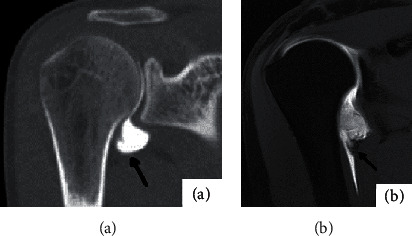
Male, 24 years old, with recurrent anterior dislocation of right shoulder for 1 year. (a) shows the coronal bone window image of CTA in the right shoulder. The shape of the axillary capsule is irregular in arrow. (b) shows the right shoulder MRA coronal T1WI-FS image. The arrow shows the inferior glenohumeral ligament with nonunion of the fibers on the humerus side, retraction of the broken end, and partial contrast agent leakage.

The inferior glenohumeral ligament was injured in 24 of the 36 afflicted shoulders. 14 cases were detected by CT arthrography, and the transverse axial bone window revealed local discontinuity of the inferior glenohumeral ligament, reduced tension, retraction of the broken end, and leakage of some contrast agent through rupture on CT arthrography (Figure 1(a)), but 3 cases without the leakage of contrast agent (Figure 2(a)). 22 cases were detected by MR arthrography, and t1WI-FS in transverse and coronal positions showed good results (Figures 1(b) and 2(b)), showing local discontinuity of the inferior glenohumeral ligament, reduced tension, retraction of the broken end, and leakage of some contrast agent through rupture.

### 3.2. Anterior Inferior Labrum Injuries

**Figure 3:**
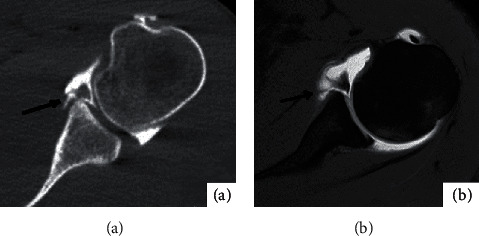
Male, 29 years old, with recurrent anterior dislocation of left shoulder for half a year. (a) shows the bone window image in the transverse axis of the left shoulder CTA, and (b) shows the T1WI-FS image in the transverse axis of the left shoulder MRA. All the black arrows show local nonunion of the anterior lower lip and contrast agent entering the fissure.

There were 32 anterior inferior labrum injuries in the 36 affected shoulders, all of which were Bankart injuries. The transverse axial bone window revealed good outcomes (Figure 3(a)), with morphological alterations, nonunion or abnormalities of the anterior and inferior labial lips, local cracks, and contrast agent entrance being the most common symptoms. However, MR arthrography found 30 cases, and the transverse t1WI-FS showed promising results (Figure 3(b)). The results of MR arthrography for anterior and lower labial injuries were comparable to CT arthrography.

### 3.3. Bone and Cartilage Injury

**Figure 4:**
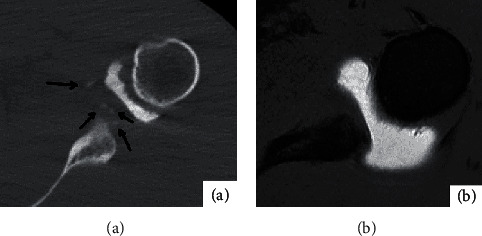
Male, 27 years old, anterior dislocation of left shoulder for 3 months. (a) shows the transverse bone window image of CTA on the left shoulder. Black arrow shows that several high-density bone fragments can be seen beside the lower margin of the glenoid cavity. (b) shows the T1WI-FS image in the transverse axis of the left shoulder MRA, and no obvious bone fragments are seen beside the lower margin of the glenoid cavity.

**Figure 5:**
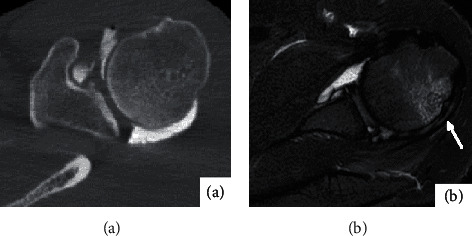
Male, 23 years old, anterior dislocation of left shoulder for 1 month. (a) shows the transverse bone window image of CTA on the left shoulder. No obvious abnormalities were found in the morphology and density of the humerus. (b) shows the PDW-FS image of the left shoulder on the horizontal axis of MRA, and the signal of the patellar bone marrow edema outside and above the humeral head was shown in the white arrow.

**Figure 6:**
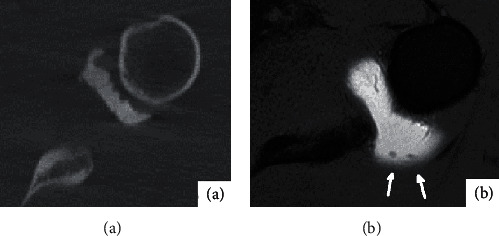
Male, 19 years old, with anterior dislocation of the left shoulder for 2 months. (a) is the bone window image of CTA transverse axis of the left shoulder. No obvious free cartilage tablets were found in the joint capsule. (b) shows the T1WI-FS image of the left shoulder in the horizontal axis of MRA. White arrows show two free small cartilage tablets in the joint capsule.

30 of the 36 affected shoulders had bone and cartilage injury, mainly manifested as osseous Bankart injury at the anterior lower margin of the glenoid cavity and Hill-Sachs injury at the outer upper margin of the humeral head. Eighteen fractures were detected by CT arthrography, showing local bone morphological changes; high-density bone fragments were separated and shifted (Figure 4(a)); In contrast, MR arthrography is less likely to detect fractures (Figure 4(b)).MPR three-dimensional imaging showed good results, and MR arthrography showed 8 fractures, showing local bone morphological changes; low signal bone fragments were separated and shifted; and various sequences in the horizontal axis showed good results. CT arthrography failed to show bone contusion (Figure 5(a)).There were 10 bone contusions, which all were detected by MR arthrography, showing high lamellar lipids and good lipid sequence in all directions (Figure 5(b)). There were 5 cartilage injuries, among which 0 were detected by CT arthrography (Figure 6(a)) and 5 by MR arthrography, showing gray hyaluronic defect on the articular surface. Gray-free cartilage tablets were seen in the articular cavity, and the t1WI-FS in the transverse axis showed good results (Figure 6(b)).

## 4. Discussion

Injuries to the inferior glenohumeral ligament and anterior inferior labrum are common pathological changes in anterior dislocation of the shoulder joint and are considered to be the most important causes of recurrent dislocation of the joint [2]. They can be accompanied by fractures of the external posterior humeral head and the anterior lower edge of the glenoid cavity, bone contusions, and articular cartilage injuries.

The labium is a fibrous cartilage that is dispersed around the bony joints, deepening the glenoid fossa and supporting the glenohumeral joints, comparable to the meniscus. The anterior labrum is frequently damaged by anterior dislocation or instability of the shoulder, which leads to repeated dislocation and instability of the anterior shoulder [3].

The upper glenobrachial ligament, middle glenobrachial ligament, and lower glenobrachial ligament strengthen the anterior articular capsule on the inner wall of the anterior articular capsule. The anterior inferior labrum, the anterior inferior glenoid ligament, and the anterior inferior articular capsule constitute the anterior inferior labrum-ligament complex, which is the most important functional device for the anterior stability of the shoulder [4]. In the case of anterior dislocation of the shoulder joint, the anterior inferior label-inferior glenohumeral ligament complex is the first to be injured, and the type of injury directly affects the surgical method and prognosis. For example, although the edge side of shoulder joint, after the former torn by arthroscopic shoulder joint capsule under repair, but the joint capsule body torn, humerus lateral joint capsule tearing, and severe joint capsule tearing are arthroscopic surgery taboo, you must repair by open surgery [5–8], therefore, preoperative accurate knowledge of joint capsule and dishes humerus ligament damage, the spoon lips injury situation, has important clinical value.

When below the humerus head forward dislocation, and edge of glenoid cavity before collision form shear stress, on the outside after the head of the humerus and glenoid cavity edge of former can cartilage injury, bone contusion and fracture and cartilage slices or pieces of bone into the joint cavity forming loose bodies can cause irritation and traumatic osteoarthritis secondary to again and again, so it is necessary surgery to remove loose bodies [8–10].

In this study, it was found that MRA has more advantages for lower glenohumeral ligament injuries, because MRI itself has high resolution for soft tissue and can clearly display upper, middle, and lower glenohumeral ligaments and joint capsule structures [10–13]. The 22 cases detected by MRA all had partial contrast agent leakage through the rupture opening, whereas the three cases detected by CTA had no contrast agent leakage through the rupture opening, which could be due to uneven contrast agent distribution and partial fibrous adhesion between the broken ends of the torn inferior glenohumeral ligament. Following a CTA examination, shoulder joint mobility uniformizes the contrast agent distribution and separates the adhesion broken ends. As a result, during an MRA examination, contrast agent can be seen leaking through the rupture site. For anterior inferior labial injuries, both CTA and MRA have high detection rates, because the main component of the labial is fibrous cartilage, and normally, the transverse axis presents triangular low density or uniform low signal. When the labial is torn, the morphology of the labial changes and contrast agent enters into the tear, which is not difficult to determine [12–18]. Because of its thin-layer scanning and 3D reconstruction functions, CTA is more likely to discover small bone fragments than MRA, and white high-density bone fragments are easier to see on CT pictures than black low-density bone fragments on MRI images. Bone marrow edema is the most common symptom of bone contusion. The compression fat sequence of MRI (pdw-fs or t2wi-fs) has unique benefits for the diagnosis of bone marrow edema due to its high sensitivity and specificity [18–20] and is the best approach for the detection of bone marrow edema. As a result, MRA outperforms CTA when it comes to detecting bone marrow edema. The implementation of a predictive control method based on deep learning [21, 22] onto the orthopedic diagnosis of patients may prove to be an effective technique in the future. Due to the limitation of resolution, CTA shows poor cartilage structure, and the free cartilage slices with equal density are easy to be covered under the background of white contrast agent in the joint cavity. However, due to the good soft tissue resolution, hyaluronic cartilage rich in water shows equal or slightly higher gray signals in MRI images. MRA can directly observe articular cartilage. When cartilage has cracks or defects, the entry of high signal contrast agent will set off the contrast of the wound surface, and the gray-free cartilage tablets will not be difficult to show under the contrast of contrast agent in the articular cavity. Therefore, MRA is more advantageous than CTA for cartilage injury.

## 5. Conclusion

In conclusion, for the diagnosis of anterior dislocation of shoulder, MRA is superior to CTA. MRA plus CT examination is the best combination of preoperative imaging examination. If the patient cannot undergo MRA examination, CTA examination can be used as an alternative to provide some valuable diagnostic information.

## Figures and Tables

**Table 1:** Comparison of CT arthrography, MR arthrography, and surgical results on detecting lesions of anterior dislocation of shoulder.

Modalities	Inferior GHL injury	Bankart lesion	Bone and cartilage lesion		
			Fracture	Bone contusion	Cartilage injury
CTA	14	27	18	0	0
MRA	22	30	8	10	5
Surgical results	24	32	18	0	5
*P* value	0.008	0.423	<0.001	<0.001	0.011
Chi-square value	7.111	0.642	13.846	20.000	6.400

GHL = inferior glenohumeral ligament, CTA = computed tomography arthrography, and MRA = magnetic resonance arthrography. Table 1 shows that for lower glenohumeral ligament injury, fracture, bone contusion, and cartilage injury, CT arthrography and MR arthrography showed statistically significant differences (*P* < 0.05), while for Bankart injury of the anterior lower labial, CT arthrography and MR arthrography showed no statistically significant differences (*P* > 0.05).

## Data Availability

Data available on request from the authors due to privacy/ethical restrictions.
